# A Unique Case of Empyema Secondary to Amoebic Liver Abscess

**DOI:** 10.7759/cureus.1377

**Published:** 2017-06-21

**Authors:** Adeel Nasrullah, Shujaul Haq, Haider Ghazanfar, Abu Baker Sheikh, Aisha Akhtar, Rizwan Zafar, Amara Nasir, Faizan Shaukat, Ali H Abbas

**Affiliations:** 1 Department of Internal Medicine, Shifa International Hospital; 2 Surgery, Texas Tech Health Sciences Center Lubbock; 3 Department of Cardiology, Shifa International Hospital; 4 Shifa College of Medicine, Shifa International Hospital

**Keywords:** empyema, liver abscess, entamoeba histolytica, trophozoites, treatment failure, extraintestinal manifestation

## Abstract

An amoebic liver abscess is an extraintestinal manifestation of amoebiasis that can present with complaints such as right upper quadrant pain and fever. It might not necessarily be associated with abdominal complaints and can have many other atypical presentations. It may present with lung diseases, cardiac diseases, or brain abscesses. We present a case of a patient with empyema secondary to amoebic liver abscess whose diagnosis was delayed due to an unusual presentation. A combination of radiology, serology, and therapeutic interventions led to the accurate management of the patient.

## Introduction

Amoebic liver abscess (ALA), a sequela of infection by Entamoeba histolytica, should be excluded in patients experiencing generalized pain in the right upper quadrant region, which presents with or without fever. Entamoeba histolytica infection is common in tropical countries and manifests in organs such as the intestine, or in some scenarios it may be present in the liver, pleura, and pericardium [1].

The rupture of the hepatic amoebic abscess through the diaphragm can lead to involvement of the pleura. In addition, hematogenous spread of the trophozoites, lymphatic spread, inhalation of cysts, or sympathetic activation can all be other causes [2]. This results in a pleural effusion. Among all the complications of amoebic abscess of the liver that were studied, the majority of them were inflammatory reactions of thoracic structures like pleural effusion, pneumonitis, and rupture through the diaphragm, involving in the order of frequency the following: the airways, the pleural cavity, and the pericardium [3]. Approximately, pleuropulmonary complications occur in 10% of patients with amoebic liver abscesses. Following are some of the risk factors that can cause pulmonary amoebiasis: malnutrition, chronic alcoholism, and atrial septal defect with left-to-right shunt [4-5]. We report a case of empyema associated with an amoebic liver abscess.

## Case presentation

A 42-year-old male patient with a known case of ischemic heart disease (IHD) and hypertension presented to the emergency department with fever and shortness of breath (SOB) for the past three weeks. The SOB was associated with a cough and left lower chest pain that exacerbated with breathing. There was no history of hemoptysis, weight loss, urinary or bowel complaints or yellow discoloration. The patient was previously started on antituberculous treatment (ATT) in a local hospital after he reported there with a low-grade fever, SOB, night sweats, and chest pain. On examination, the patient was in acute distress with SOB; pallor was seen. His pulse was 80 beats per min, the SpO2 was 87%, the blood pressure (BP) was 115/80 mmHg, the temperature was 103 F, and the respiratory rate was 25 breaths/min. On chest examination, there were decreased breath sounds, in the middle and lower zones on the left side. The laboratory investigations are presented in Table 1.

**Table 1:** Laboratory investigations AST - aspartate transaminase, ALT - alanine transaminase.

Laboratory Investigation	Value
Hemoglobin	11.72 g/dl
Hematocrit	36.8 %
White Blood Cells	16400/ul
Platelets	448,000/ul
AST	26 units/L
ALT	17 units/L
C-reactive Protein	386 mg/L
Serum Sodium	138 mEq/L
Serum Potassium	3.7 mEq/L
Serum Chloride	99 mEq/L
Serum Bicarbonate	19 mEq/L
Serum Creatinine	0.66 mg/dl
Serum Blood Urea Nitrogen	12 mg/dl
Serum Glucose	103 mg/dl

**Figure 1:**
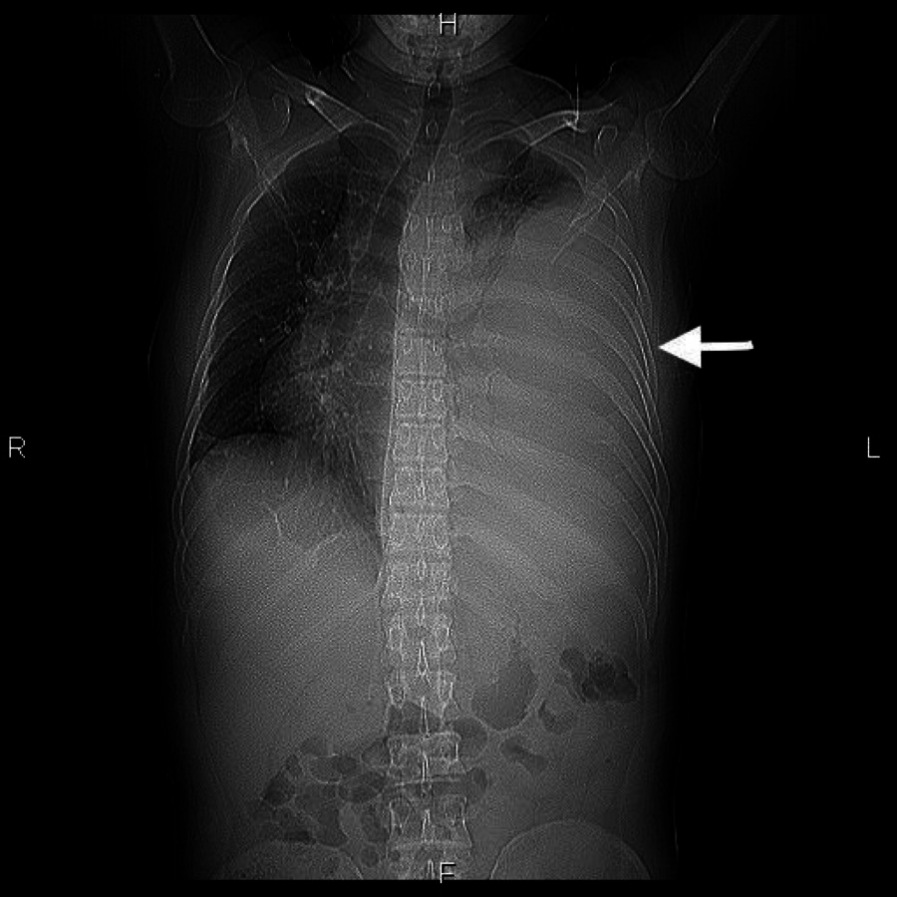
Chest X-ray showing a large left-sided pleural effusion

A chest X-ray showed a large left-sided pleural effusion. This is presented in Figure 1.

**Figure 2:**
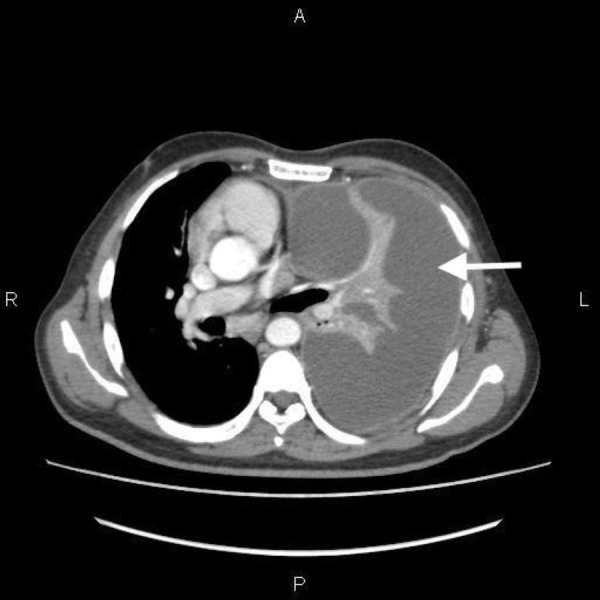
Computed tomography scan of the chest showing a large left-sided multiloculated pleural effusion with collapsed left lung

An emergency computed tomography (CT) scan of the chest was performed, which revealed a large left-sided multiloculated pleural effusion with severe compression collapse of the left lung. This is presented in Figure 2.

**Figure 3:**
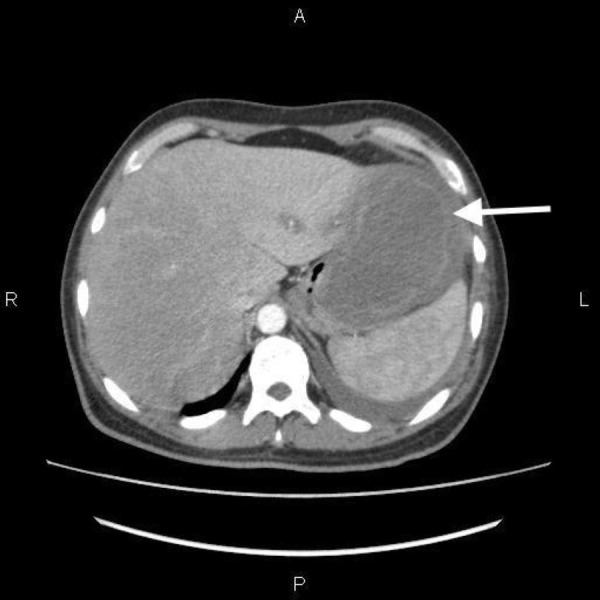
Computed tomography scan of the abdomen showing a large left-sided sub-phrenic collection involving the left lobe of the liver

A large left-sided subphrenic collection was also noted. This is presented in Figure 3.

A chest tube was inserted with the goal of draining the pleural fluid collection, but it only resulted in a mild reduction of the pleural fluid collection with a slight improvement of left upper lobe aeration.

The ATT was stopped after three acid-fast bacilli smears were found to be negative, and empiric broad-spectrum antibiotic therapy was initiated. Despite the medication regimen, the patient continued to spike fevers of about 102–104 °F. Cardio-thoracic surgery consult was obtained. Video-assisted thoracoscopic surgery (VATS) was performed. On VATS, decortication and drainage of loculated pockets of pleural fluid were performed. Pleural fluid analysis revealed inflammatory infiltrate with occasional pus cells. Gram stain did not show any organisms. The patient underwent ultrasound-guided aspiration and drainage of the subphrenic abscess, but it failed to resolve the patient’s symptoms. The ultrasound also showed hepatomegaly, a subphrenic collection involving the left lobe of the liver, and mild compression of the stomach. General surgery consult was obtained after which a laparotomy was advised.

Laparotomy was undertaken and it discovered a walled-off abscess cavity at the inferior surface of the left lobe of the liver that extended up to the subdiaphragmatic space and which contained an anchovy paste-like fluid about 1 liter in quantity. The patient’s fever resolved after the abscess was drained. Based on clinical suspicion, amoebic serology was obtained, which was positive. The patient continued to improve clinically and the postoperative course was uneventful. The patient was discharged on antibiotics with a follow-up plan of care.

## Discussion

Amoebic liver abscesses occur most commonly in the age group of 20–45 years and have been noted infrequently in the extremes of ages [4], with an adult male to female ratio of 10:1 [5]. Our case is of a 42-year-old male patient who presented with the complaint of fever and SOB for the past three weeks.

The diagnosis of amoebic liver abscess is sometimes difficult since its clinical manifestations are highly variable, like in our patient who presented with a left lower chest pain, intermittent high-grade fever, and cough; in spite of not having symptoms like right upper quadrant abdominal pain, jaundice, weight loss, or hemoptysis, the patient still had the disease.

We report a case of left pleural empyema secondary to an amoebic liver abscess that was misdiagnosed as tuberculosis. Pleuro-pulmonary amoebiasis is easily confused with other illnesses, and it is treated as pulmonary TB, bacterial lung abscess, and carcinoma of the lung [5]. Aspiration and drainage of pus from thoracic empyema may be needed for confirmation and therapeutic purposes. Also, it has been recommended that amoebic liver abscess be treated with metronidazole or tinidazole plus a luminal amoebicide (eg. paromomycin or iodoquinol) even if the intestinal infection is not documented [6-7].

Imaging techniques such as ultrasound, computed tomography (CT), and magnetic resonance imaging (MRI) have excellent sensitivity for the detection of a liver abscess and were used with our patient, but these techniques cannot distinguish amoebic abscesses from pyogenic abscesses or necrotic tumor. The diagnosis of an amoebic liver abscess is confirmed with either serologic or antigenic testing. It can also be coupled with stool microscopy and antigen testing of the stool, with or without evaluation for the parasite in the hepatic abscess fluid [8].

Due the combination of findings in the imaging studies like hepatomegaly, pleural effusion with thick loculated collection, obliteration of costophrenic and costodiaphragmatic angles, left subdiaphragmatic collection, and involvement of the left lung which suggested an empyema of the left lung, the patient was treated with a chest tube and percutaneous liver abscess drainage. But it was a surgical intervention, a laparotomy, which helped discover a well walled-off abscess cavity at the inferior surface of the left lobe of the liver that extended up to the subdiaphragmatic space that contained an anchovy sauce-like fluid, about 1 liter in quantity. Following surgical drainage, the fever improved dramatically as he continued to be under observation.

Some literature indicates that percutaneous needle aspiration or catheter drainage may be helpful for large abscesses (over 5-10 cm), in particular, if the diagnosis is uncertain, if there is an initial lack of response, or if a patient is very ill, suggesting impending abscess rupture, while some authors have had more liberal thresholds of maximum diameter >10.7 cm and intervention only in the absence of response to drugs [9]. Therefore, in cases that fail to respond to the conventional treatment, interventions such as needle aspiration, catheter drainage, or surgical interventions can be employed as required.

## Conclusions

Amoebic liver abscesses can present with a varied set of complaints and there might be no abdominal symptoms at all. The patient could have avoided these complications if the diagnosis had been made earlier. The combination of clinical manifestations with a serial imaging might give us a clue that can lead to the timely diagnosis and prompt management of amoebic liver abscess, avoiding further complications like amoebic empyema.
